# Dissecting the sterility phenotype in gene edited *Drosophila suzukii* pgSIT males

**DOI:** 10.1038/s41598-025-88598-w

**Published:** 2025-01-31

**Authors:** Avery D. Witherbee, Stephanie Gamez

**Affiliations:** Agragene Inc., St. Louis, MO USA

**Keywords:** Biotechnology, Molecular engineering, Genetics

## Abstract

*Drosophila suzukii* is an invasive pest that affects many fresh, soft-skinned fruits such as strawberries and blueberries. To combat this pest, growers use various methods including insecticide sprays, biological control, and sanitation practices. However, these methods are becoming increasingly ineffective against *D. suzukii* due to increased resistance against insecticides and increasing labor costs. Sterile Insect Technique (SIT) has been used successfully to control many agricultural pests, but the use of irradiation, sex sorting, and the requirement to scale these insects make it costly to implement. pgSIT (precision guided Sterile Insect Technique) is a novel and efficient way to generate sterile males through genetic engineering and overcomes the drawbacks of traditional SIT. pgSIT has been implemented in multiple Dipteran insects, including *D. suzukii*, and has been shown to suppress wild insect populations. To further characterize sterile pgSIT males, we evaluated their fertility capacity, lack of mature sperm, and ability to induce a mating refractory period in *D. suzukii* wildtype females. In this study, we found that pgSIT sterile males do not produce mature sperm and can induce a refractory mating period in wildtype females. Furthermore, sperm DNA is not detected in the reproductive tracts of pgSIT-mated female reproductive tracts. These findings further support the penetrance of the pgSIT technology in *D. suzukii* and provide further supporting data to governing regulatory bodies in their evaluation of this technology.

**Supplementary Information:**

The online version contains supplementary material available at 10.1038/s41598-025-88598-w.

## Introduction

Spotted wing drosophila (*Drosophila suzukii*, SWD) is a significant invasive species, originating from eastern and southeastern Asia^1^. It was first spotted in North America and Europe in the late 2000s and their geographic spread has only increased^1,2^. *D. suzukii*, unlike other *Drosophila* species, are considered crop pests due to their attraction to fresh fruits rather than overripe, rotting fruits^2^. Many soft-skinned agricultural crops such as strawberries, cherries, and raspberries are affected by *D. suzukii* females equipped with large, serrated ovipositors which enable them to lay eggs in the fresh fruits^1,2^. This allows the eggs to develop into larvae which feed on the fruits, causing them to become damaged^1,2^. The fruit penetrating ovipositor can also lead the punctured fruits to be more susceptible to other insects, fungi, and bacteria^3^. These damaged fruits result in decreased crop yields and economic loss^1,4^.

Management of this pest is key to addressing the economic losses growers face. Often, a combined approach using different strategies, Integrated Pest Management (IPM), is deployed. Common IPM approaches include cultural practices (i.e. pruning, mass trapping, fruit removal and sanitation), biological practices (i.e. nematodes, microorganisms, parasitoids), and chemical practices (conventional and organic insecticides)^5^. Despite all these practices, growers rely on synthetic chemicals to reduce SWD populations. Although very effective at killing SWD, insecticides are broad spectrum and affect non-target insects including pollinators. Growers are faced with using multiple spray applications to combat this pest, but insecticide resistance develops after repeated exposure^6,7^. Coupled with the high reproductive rates and short generation time of SWD, evolution against insecticides arises rapidly. Growers are now moving towards using more environmentally friendly practices due to the increasing demands for cleaner environments and safe food. Therefore, growers need novel and effective tools for managing SWD.

The Sterile Insect Technique (SIT) is an effective pest control tool which relies on the use of sterile insects for the management or suppression of target insect pests. SIT has been used for over 70 years and it is responsible for successful eradication programs of some agricultural pests including the screw-worm fly (*Cochliomyia hominivorax*)^8^, Mediterranean fruit fly (*Ceratitis capitata*)^9^, tsetse fly (*Glossina austeni*)^10^, and the Mexican fruit fly (*Anastrepha ludens*)^11^. Implementing SIT requires mass rearing of the target insect, sex-sorting, and complete sterilization by irradiation^12^. The levels of radiation must be high enough to sterilize the insect, but not severely impact survival and ability to mate^13–15^. To overcome irradiation-associated fitness costs, multiple releases of these sterile insects must be performed to achieve population suppression. Despite the numerous successful SIT programs, SIT can be expensive to implement depending on the insect target. For example, SIT is challenging to implement in Lepidopteran pests because of the holokinetic structure of their chromosome that makes them resistant to radiation^16^. In these Lepidopteran insects, the irradiated parents, which are partially sterile, produce completely sterile F1 offspring. This characteristic adds on to the operational costs of mass-rearing Lepidopteran sterile males. In other insect targets like Tephritids, the USDA-APHIS determined that an Oriental Fruit Fly SIT program costs outweigh the benefits in reducing eradication. Costs associated with operating the Hawaii rearing facility and Califonia eclosion facility, labor, shipping, and releases made the program expensive^17^. This is one of the reasons why governments often sponsor and maintain these short term SIT eradication programs^18,19^. These costs make it difficult for governments and private companies to establish/maintain SIT programs for pest management.

Recently, pgSIT (precision-guided Sterile Insect Technique) has been developed to overcome the costs and drawbacks of traditional SIT^20–22^. This novel form of SIT uses CRISPR-based gene editing to simultaneously disrupt genes essential for female viability and male fertility to result in the production of sterile males^20^. A simple genetic cross is required between the Cas9 and guide-RNA (gRNA) breeding lines to produce completely sterile males. No female offspring result after this cross and thus skips the costly sex sorting step prior to release. Both Cas9 and gRNA breeding lines scale separately and are only mated together when sterile males need to be produced—a binary system providing a level of control^20–22^. This technology has been demonstrated in several insects and provide little to no fitness cost on the breeding lines as well as the sterile male itself^20–22^. pgSIT is a powerful technology that can be implemented in an IPM strategy for the control of agricultural pests. To date, this technology is characterized as a “biopesticide” and regulated as a pesticide by the United States Environmental Protection Agency (US-EPA) and as a “plant pest” by the United States Department of Agriculture (USDA). Before pgSIT can be registered in the U.S., a rigorous evaluation process by both agencies must take place to assess the risk of using a biotechnology product to protect agricultural crops.

In this study, we seek to further dissect the sterility phenotype of the pgSIT sterile males in SWD for the small fruit industry stakeholders and U.S. regulating agencies. Here, we demonstrate a lack of genetic material passed on from pgSIT males to wildtype females after mating and the subsequent induced refractory mating period. We find no negative fitness costs associated with fertility in the Cas9 and gRNA breeding lines, further lending support to the scalability of this technology and a critical component in SIT programs. These findings provide insight into the unique aspects of pgSIT for insect population control and demonstrate that pgSIT SWD males behave like wildtype SWD males in terms of inducing mating responses in mated females. The data produced in this study will help regulatory bodies assess the potential risk of using pgSIT as a viable control method for suppressing SWD and other pest populations while minimizing the environmental footprint of pest control measures.

## Results

### Generation of completely sterile *D. suzukii* males

**Fig. 1:**
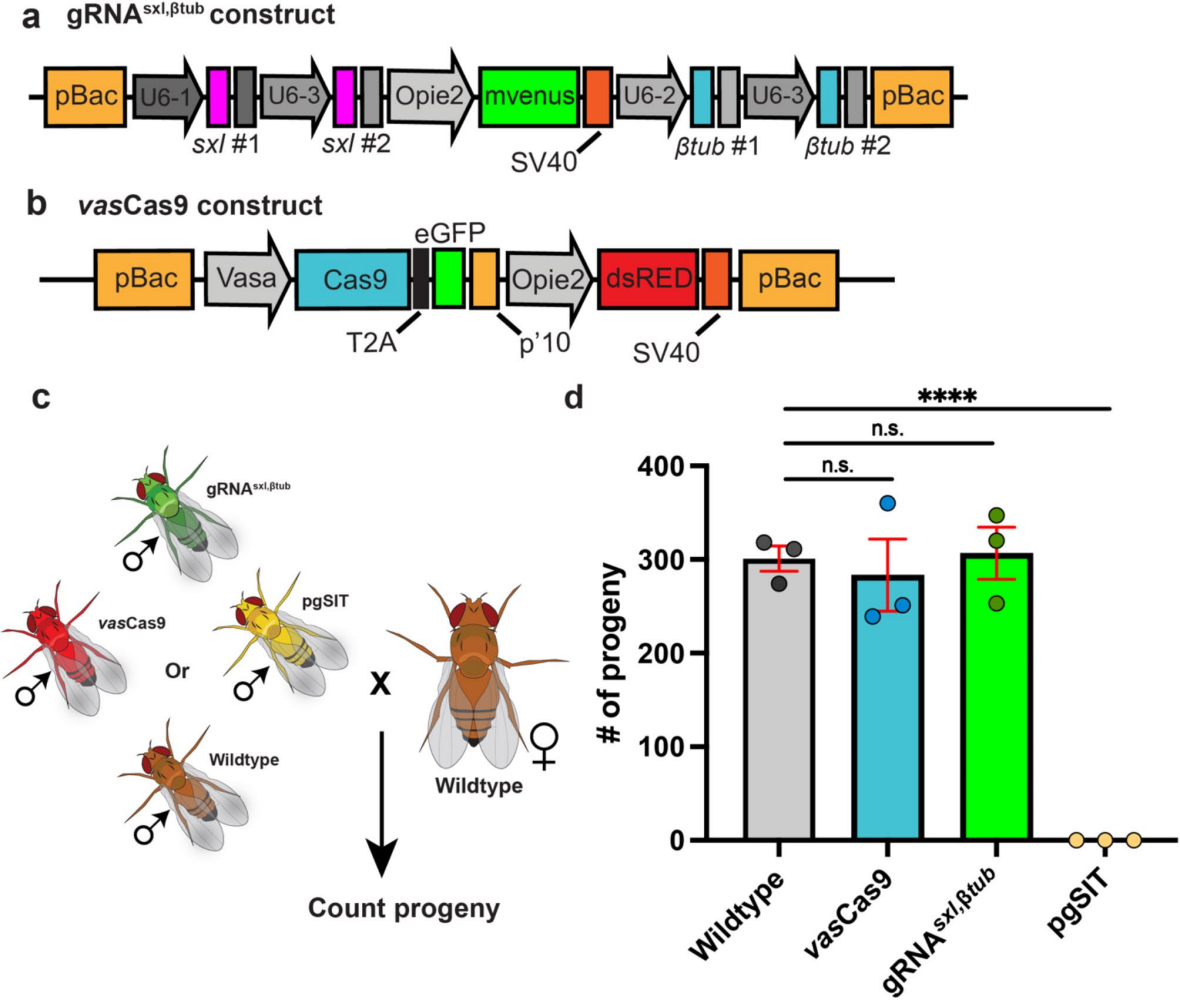
pgSIT *Drosophila suzukii* males are 100% sterile. (**a**) A schematic of the gRNA^sxl, βtub^ construct used to generate the gRNA line. This line is tagged with a green-fluorescent genetic marker. (**b**) Schematic representation of the *vas*Cas9 construct used to generate the Cas9 line. This line is tagged with a red fluorescent genetic marker. (**c**) The crossing scheme used to assess the fertility of males from wildtype, *vas*Cas9, gRNA^sxl, βtub^, and pgSIT strains. Males from each strain were separately outcrossed to wildtype females. Progeny from these crosses were used to assess fertility. (**d**) pgSIT males are completely sterile compared to wildtype, *vas*Cas9, and gRNA^sxl, βtub^ outcrosses. No significant differences were observed when comparing *vas*Cas9 and gRNA^sxl, βtub^ offspring data to wildtype. The average number of offspring for each strain is plotted as colored bars. The colored circles indicate the number of offspring in each replicate. The red vertical bars indicate ± SEM. Statistical significance of means was evaluated using unpaired t-test. (*p* > 0.05^ns^, *p* < 0.0001^****^).

To assess the fertility of *vas*Cas9, gRNA^sxl, βtub^, pgSIT, and wildtype adult males, males from each strain were independently mated with virgin wildtype females and resulting offspring were counted (Fig. 1C). As expected, pgSIT males produced no offspring (0 ± 0.0 [mean ± SEM]; total number of offspring over three replicates = 0) compared to wildtype males (301 ± 19.3 [mean ± SEM]; total number of offspring over three replicates = 903), confirming pgSIT sterility (Fig. 1D). *vas*Cas9 (283 ± 54.4 [mean ± SEM]; total number of offspring over three replicates = 850) and gRNA^sxl, βtub^ (306 ± 39.5 [mean ± SEM]; total number of offspring over three replicates = 920) males produced offspring comparable to wildtype males, suggesting similar fertility among the modified and unmodified strains (Fig. 1D). All raw data is presented in Supplemental Table S1.

### pgSIT sterile ***D. suzukii*** males induce a refractory mating period in wildtype females

**Fig. 2:**
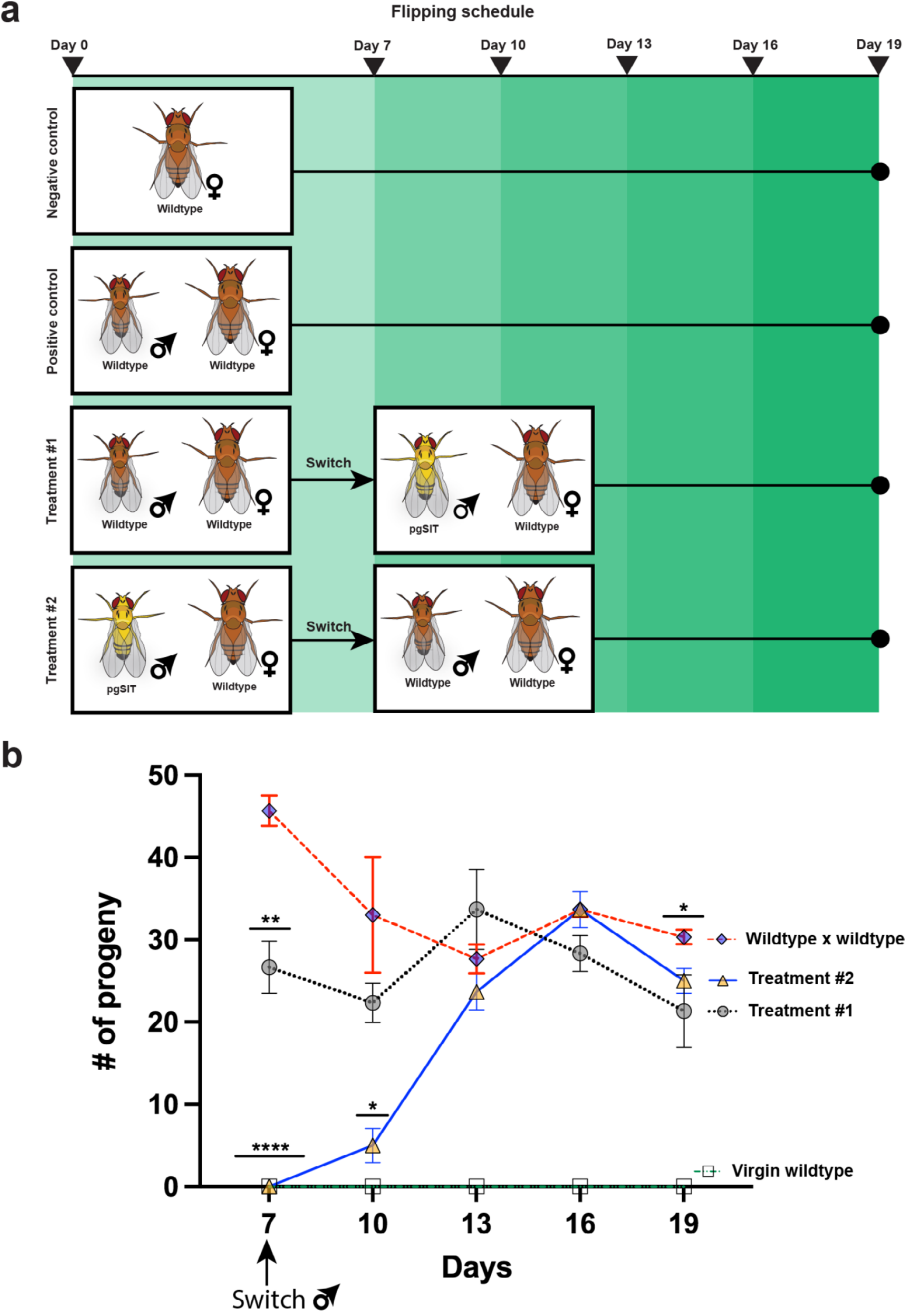
pgSIT sterile males induce a refractory mating period in wildtype females. (**a**) Schematic of the re-mating experimental set up. The negative control consisted of only virgin wildtype females and the positive control consisted of wildtype males mated with wildtype virgin females. Treatment 1 initially mated wildtype males with wildtype virgin females then switched after 7 days to pgSIT males mating with the wildtype females. Treatment 2 initially mated pgSIT males with wildtype virgin females then switched to wildtype males after 7 days. (**b**) Progeny counts of the different re-mating treatments and controls. The negative control represented by white squares and a green dotted line produced no offspring. The positive control represented by blue diamonds and a red dashed line produced offspring throughout the duration of the experiment. Treatment 1 represented by gray circles and a black dotted line produced offspring throughout the experiment, reaching its peak at 13 days then declining afterwards. Treatment 2 represented by yellow triangles and a blue line produced no offspring when first mated with pgSIT and after the switch to wildtype males, began to increase offspring production. The colored vertical bars indicate ± SEM. Statistical significance of means was evaluated using unpaired t-test. (*P* ≤ 0.05^*^, *P* ≤ 0.01^**^, *P* < 0.0001^****^).

Evaluating the remating ability of wildtype females after the initial mating provides a glimpse into the sexual competitiveness of the sterile males. To assess if pgSIT males could induce a refractory mating period in wildtype females, a series of mating crosses were performed, and the resulting offspring were counted (Fig. 2A). Throughout the duration of this experiment, the negative control (virgin wildtype females only) produced no offspring (Fig. 2B). The positive control (wildtype males paired with wildtype females) produced offspring throughout the experiment.

Throughout the experiment, Treatment 1 (wildtype males switched to pgSIT males) experienced an increase in the average number of offspring between days 10 and day 13 (Fig. 2B). After day 13, Treatment 1 experienced a downward trend until the experiment stopped at day 19. The pgSIT males in Treatment 2 (pgSIT males switched to wildtype males) induced refractoriness in wildtype females after the initial 7-day mating period. The average number of offspring went from 0 to 5, a slight increase in comparison to the offspring averages from Treatment 1 and the positive control (Fig. 2B). This clearly demonstrates a delay in the number of offspring produced when wildtype females initially mate with sterile pgSIT males. Treatments 1 and 2 increased offspring production after the second mating period (day 10) while the positive control decreased offspring production. After the third mating period (day 13), Treatment 1 experienced a downward trend in production of offspring. Both the positive control and Treatment 2 offspring production fluctuated up then down after the third mating period. At the end of the experiment, both Treatments 1 and 2 experienced a downward trend. All raw data is present in Supplemental Table S2.

### pgSIT males induce normal physiological ovary changes in females

**Fig. 3:**
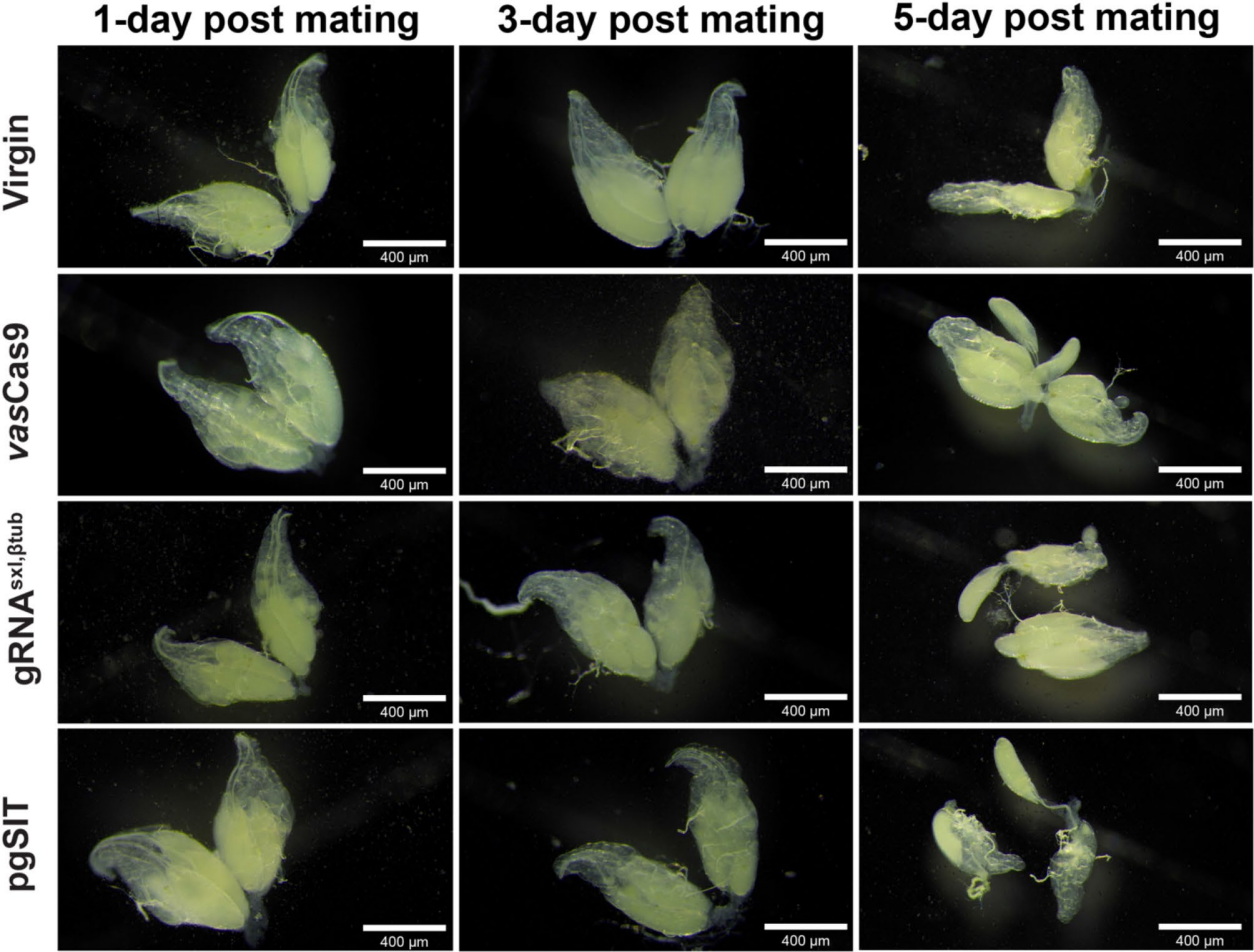
Dissections of mated wildtype females after 1-, 3-, and 5-days post mating. Virgin wildtype females were paired with either no males, *vas*Cas9, gRNA^sxl, βtub^, or pgSIT males. After 1-, 3-, and 5- days post mating, ovaries were dissected from females to visualize the oocytes within. Scale bars in the bottom right corner of the images represent 400 μm.

Crosses between virgin females and *vas*Cas9, gRNA^sxl, βtub^, or pgSIT males were done and ovaries were collected and imaged after 1-, 3-, and 5-days post mating (Fig. 3). The negative control, virgin unmated-females, was also used to visualize unmated oocyte development in virgin females (Fig. 3). After 1-day post mating, oocyte development can be seen in ovaries of females mated with *vas*Cas9, gRNA^sxl, βtub^, and pgSIT. Some oocyte development is also seen in virgin female ovaries. In the 3-day post mating, a similar number of oocytes are seen in all mated and unmated ovaries. In the 5-day post mating, three oocytes were seen in the virgin ovaries compared to the ovaries from females mated to *vas*Cas9 and gRNA^sxl, βtub^ (Fig. 3). In the samples where females mated to males of *vas*Cas9, 8 visible oocytes were seen. In gRNA^sxl, βtub^ matings, 7 visible oocytes were seen. Dissected ovaries from females mated with pgSIT males had one ovulating ovary and an early-stage oocyte in the second ovary (Fig. 3).

### pgSIT males do not transfer genetic material

**Fig. 4:**
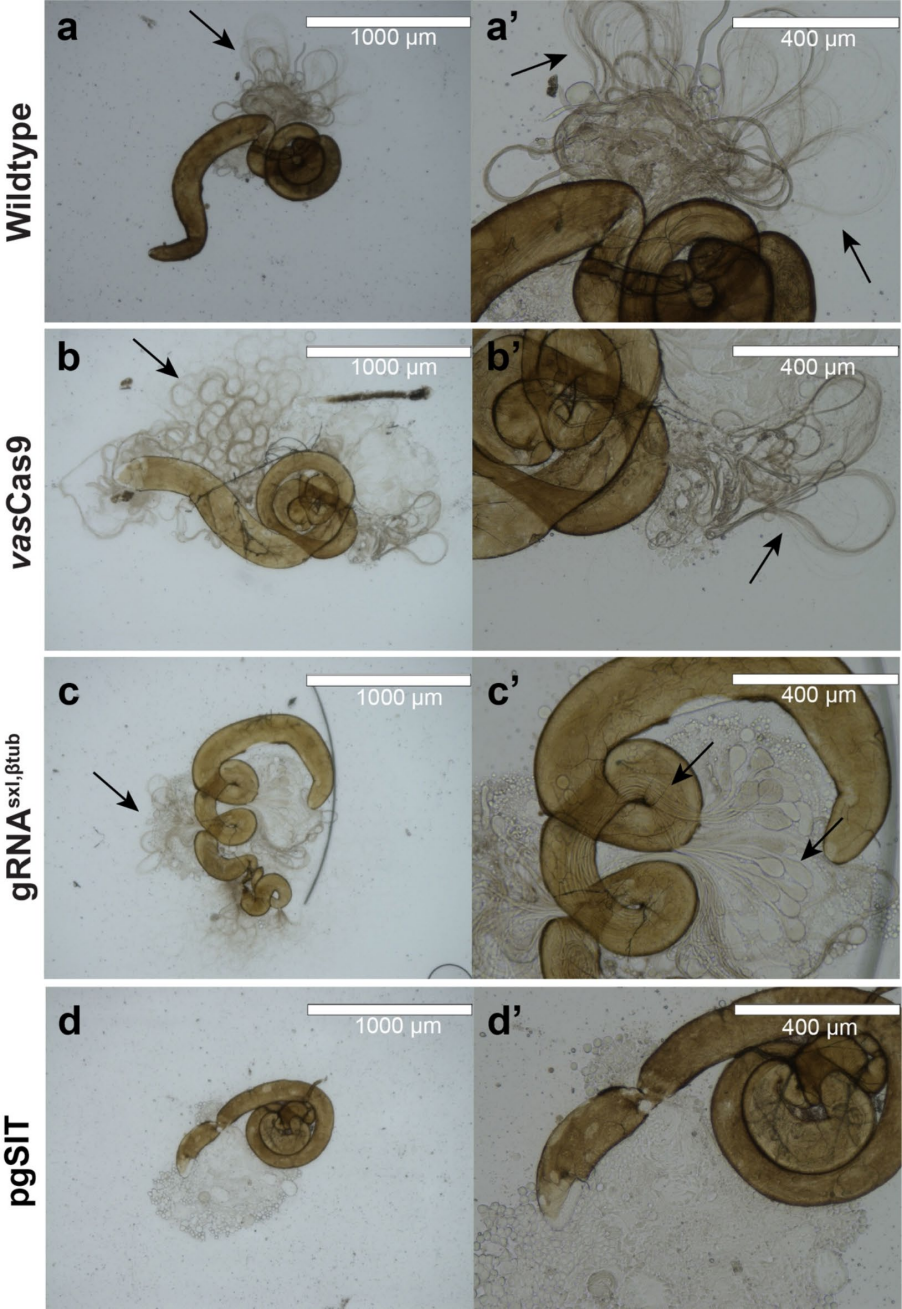
pgSIT sterile males do not produce mature sperm. Images of dissected male testis from wildtype (**a**), *vas*Cas9 (**b**), gRNA^sxl, βtub^ (**c**), and pgSIT (**d**) strains. The magnified images of wildtype (a’), *vas*Cas9 (b’), and gRNA^sxl, βtub^ (c’) testis show mature sperm flow out of the punctured tissue. The close-up image of the pgSIT male testis demonstrates the lack mature sperm (d’). Black arrows point to hair-like structures that are the mature sperm. Scale bars on the top right of each image represent 1000 μm (**a**-**d**) and 400 μm (a’-d’).

**Fig. 5:**
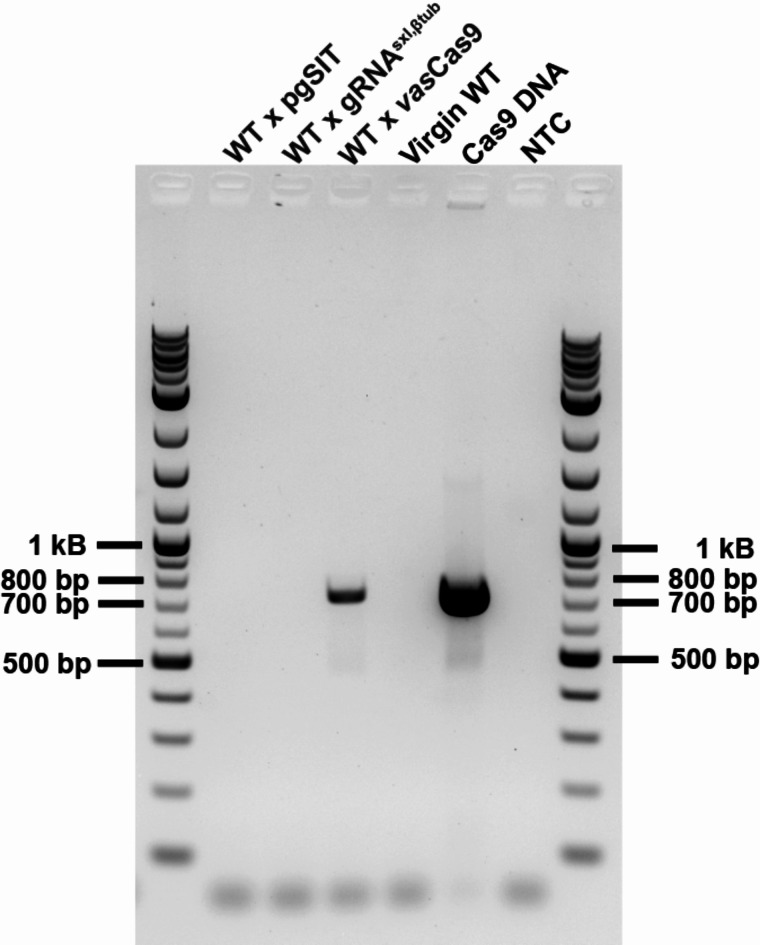
pgSIT sterile males cannot transfer sperm in wildtype matings. Reproductive tracts dissected from mated females were used to assess DNA transfer from males. Using a primer set that amplifies a 731 bp segment of the *vas*Cas9 transgene, PCRs were performed to detect transfer of sperm from wildtype females mated to *vas*Cas9 males. The *vas*Cas9 amplicon was detected in the reproductive tracts of females mated to *vas*Cas9 males. The positive and negative controls behaved as expected. No *vas*Cas9 amplicon was detected in reproductive tracts of females mated with pgSIT sterile males and gRNA^sxl, βtub^ males, nor in virgin females. A 1 kB ladder flanks the ends of the gel.

To confirm the basis of sterility, testes were dissected and imaged from 4-day old *vas*Cas9, gRNA^sxl, βtub^, pgSIT, and wildtype males. The testes of *vas*Cas9, gRNA^sxl, βtub^, and wildtype males were visibly larger than pgSIT males and contained mature sperm (Fig. 4). In pgSIT males, the testes were smaller and did not have mature sperm inside. All testes were manually punctured to confirm no mature sperm flowed out (Fig. 4). PCR was used to molecularly confirm (with the presence of the Cas9 amplicon) the transfer of sperm in mated female reproductive tracts. DNA from Cas9 males was detected in the reproductive tracts of wildtype females who mated with Cas9 males (Fig. 5). No pgSIT DNA was detected in the reproductive tracts of wildtype females who mated with pgSIT males (Fig. 5). No Cas9 amplicon was detected in the non-template control as well as the reproductive tracts of virgin wildtype females and females who mated with gRNA^sxl, βtub^ males. As expected, the Cas9 amplicon was detected in the positive control (Cas9 gDNA). Unfortunately, no gRNA^sxl, βtub^ amplification was possible in the reproductive tracts of females mated with gRNA^sxl, βtub^ males (data not shown). This is likely due to extremely low levels of sperm transferred after mating. Therefore, this data was excluded from the results.

## Discussion

pgSIT is a powerful technology that utilizes CRISPR-Cas9 technology to precisely sterilize insect males for insect population control^20–22^. This technology is of critical importance in the agricultural industry as part of a grower’s goal to reduce the usage of synthetic chemicals for pest control.

Agragene has developed an improved form of pgSIT in *D. suzukii* and seeks to obtain regulatory approvals to conduct field trials and ultimately commercialize this product in the US. As a biopesticidal product which is derived from a well-known plant pest (SWD), both the United States Department of Agriculture (USDA) and the U.S. Environmental Protection Agency (US-EPA) have shared regulatory oversight governing the movement, confined testing, distribution and environmental release of this product. This shared jurisdiction and oversight of this unique biocontrol product was recently described as part of the White House’s efforts to update, streamline, and clarify their regulations and oversight for biotechnology products^23^. In particular, the USDA’s role is to ensure that the novel modified fly does not present a ‘plant pest risk’ while the USEPA’s focus is to ensure that the pesticidal product will not cause “unreasonable adverse effects to human health and the environment”. Moreover, the EPA has been granted authority to set tolerances for these products which further ensures there is a “reasonable certainty that no harm will result from aggregate exposure to the pesticide residue.” To register and commercialize a biopesticide, the EPA and USDA must complete comprehensive and rigorous assessments of the product by utilizing existing and newly generated data—for example, toxicology studies, in vitro digestibility studies, non-target organism evaluations, persistence studies, and physicochemical measurements–as well as information from the literature. Additional (or less) information may also need to be submitted to the various agencies for a given product based on the modified fly’s unique risk profile and related characteristics. Lastly, it is of paramount importance to fully assess and demonstrate the efficacy of the product through a series of confined and open release evaluations in the field as part of the product development process.

To conduct field trials and obtain commercial registration in the U.S., regulatory approval must be granted from U.S. regulatory agencies including the United States Department of Agriculture (USDA) and the U.S. Environmental Protection Agency (US-EPA). Fully characterizing the modified flies and ensuring there is complete penetrance of the desired sterility trait amongst released pgSIT males is critical to evaluating efficacy and ensuring release of these novel flies does not present a environmental safety concern. Therefore, in this study, we sought to understand the sterility phenotype of the pgSIT sterile males in SWD for SWD pest control. The efficacy of pgSIT depends on the penetrance of the male sterility and female lethality phenotype (Fig. 1D). Consistent with previous pgSIT work in Drosophilds and mosquitoes, our study demonstrated 100% male sterility in pgSIT SWD males and complete female lethality in the F1 offspring of gRNA^sxl, βtub^ and *vas*Cas9 parents. When examined independently, the breeding lines have comparable fertility to wildtype flies, suggesting no fitness cost is incurred in fertility in the breeding lines. This will allow for rapid expansion of the breeding lines for pgSIT sterile male production. In addition to confirming the sterility penetrance, we found that the pgSIT sterile males can induce a refractory mating period in their wildtype mates. Insect females can undergo a refractory mating period where they do not mate again for a period of time^24–26^. The efficacy of pest control using SIT methods depend on the sexual competitiveness of the mass-reared males. Since pgSIT is a novel version of SIT, the in-field efficacy of this technology will also depend on the sexual competitiveness of the gene edited sterile males. Understanding whether the mated female undergoes a period of no mating after the initial mating (refractory mating period) is an important consideration for SIT programs^27^. The refractory mating period induced by pgSIT SWD males suggests that these males can induce these normal mating behaviors in females^28^. Our data clearly demonstrates a delay in the number of offspring produced when wildtype females initially mate with sterile pgSIT males (Fig. 2B). In addition, a downward trend in the production of offspring is seen in wildtype females (initially mated with wildtype males) as they mate with the pgSIT sterile males throughout the duration of the experiment (Fig. 2B). This data can be used to inform on release frequency to achieve maximum efficacy in the field.

An examination of dissected ovaries from mated females did not yield any obvious differences amongst the treatments. In preliminary dissections (data not shown here), ovaries dissected five days post mating with pgSIT males resulted in large bundles of oocytes. We hypothesized that these females were undergoing the hormonal and physiological changes post-mating^29,30^. However, in the current study we were unable to see these post-mating characteristics in the dissected oocytes. This could be due to the timing of dissections. It is likely the mated females laid eggs before the dissections occurred and thus appeared to have less oocytes bundled in each ovary. Indeed, we did detect more mature oocytes in the samples from females mated to *vas*Cas9 and gRNA^sxl, βtub^ males compared to the pgSIT mated female ovaries. This likely suggest fertile males were able to transfer sperm and induce egg production in mated females. In the virgin dissections, oocytes were present in the ovaries despite the lack of males in this treatment. It is well known that virgin *Drosophila melanogaster* females will lay a small number of unfertilized eggs and represents the initial prerequisite for the evolution of parthenogenesis^31^. In Fig. 3, the wildtype ovaries from the pgSIT 5-day post mating treatment seem to have an oocyte in the oviduct (ovulation), suggesting the female was preparing to lay this egg immediately before dissections occurred. Furthermore, in the reproductive tracts of mated females, we were able to detect the genetic material passed on from the *vas*Cas9 males and not in the tracts of females that mated with pgSIT males using *vas*Cas9-specific primers. If genetic material was indeed transferred from pgSIT males to females, detection of the *vas*Cas9-specific amplicon would appear after PCR. The absence of the *vas*Cas9 amplicon coincides with the lack of mature sperm in the testis of pgSIT sterile males (Fig. 4). In the gRNA^sxl, βtub^ mating, however, we were unable to obtain a signal (PCR amplicon using gRNA^sxl, βtub^-specific primers) in the DNA extracts of the reproductive tracts of wildtype females. We wonder if the gRNA^sxl, βtub^ sperm transferred was sufficient to fertilize oocytes, but below the limit of detection. Although we would have liked to have detected the gRNA^sxl, βtub^ amplicon for further evidence of our ability to detect transferred genetic material, the *vas*Cas9 PCR detection proved sufficient.

The work detailed in this study expands on the unique characteristic of the pgSIT technology: no genetic material is transferred during mating. Other novel insect biocontrol technologies such as the Release of Insects carrying a Dominant Lethal (RIDL), *Wolbachia*-infected insects, and gene drives have been proposed to combat insect pests. However, for these technologies to induce population suppression, they require the transfer of genetic material from the released male insect^32^. pgSIT has the advantages associated with genetically engineered insects without the risk of persisting and propagating in the environment. Before pgSIT can be widely used on the commercial scale, it must undergo rigorous evaluation by stakeholders, US-EPA, and USDA. Some biotechnologies, like RIDL and *Wolbachia*-based technologies, are further along in the regulatory approval process in some states as experimental use permits (EUP) for broad scale trials have been issued^33,34^. Interestingly, the release of irradiated sterile males (SIT) are not regulated by the U.S., despite the random DNA changes/mutations through DNA damage is caused by radiation^35,36^. We hope that this study provides additional evidence of the sterility phenotype penetrance, precise gene edits at target genes and the lack of genetic persistence and propagation in pgSIT sterile males. pgSIT is a powerful and precise tool that can be implemented in the grower’s IPM toolbox for insect pest control.

## Materials and methods

### Construct design and assembly

The initial development of the pgSIT strains in *D. suzukii* was originally described by Kandul et al.^22^. The *vas*Cas9 construct was obtained from Dr. Omar Akbari and characterized in Kandul et al.^37^ and was not modified prior to microinjection (Fig. 1B). In this study, we chose to further modify the triple gRNA construct targeting βTubulin 85D. To reduce the possibility of producing a functional mutation in the male-specific βTubulin at 85D (βTub85D or βTub, NCBI GeneID:108015852), we chose to design and incorporate two gRNA target sites. CHOPCHOP v3 was used to choose the βTub gRNA target sites that had minimal off-target activity. The sex lethal (*sxl*, NCBI GeneID:108016832) target sites described in Kandul et al., 2022 were used for targeting *sxl* gene to induce female lethality. For driving the expression of the gRNAs, we used the endogenous *D. suzukii* U6-1, U6-2, and U6-3 promoters^38^. The 3’-UTRs of each respective promoter were used directly after the gRNA scaffold to terminate gRNA transcription. To generate the pgSIT gRNA construct, gRNA^sxl, βtub^, containing a total of four gRNAs, we synthesized two intermediate dual gRNA constructs, Vector 11 A and Vector 11B using Genscript. Vector 11 A consists of a U6-3 promoter driving the expression of gRNA^sxl#1^ and U6-1 promoter driving gRNA^sxl#2^. Vector 11B consists of a U6-2 promoter driving gRNA^β^^tub#1^ and U6-3 driving gRNA^β^^tub#2^. We PCR amplified the *Opie2* promoter-*mVenus*-3’UTR SV40 fluorescent marker as a fragment from the gRNA construct described in Kandul et al., 2022. The three units, dual gRNA-sxl guides, fluorescent marker, and dual gRNA-βtub guides were PCR amplified as fragments and cloned via Gibson Assembly into a piggybac transformation construct in the following order: gRNA^sxl#1^- gRNA^sxl#2^ -fluorescent marker- gRNA^β^^tub#1^- gRNA^β^^tub#2^. The schematic map of the assembled gRNA construct, gRNA^sxl, βtub^, is presented in Fig. 1A.

### Development of pgSIT *D. suzukii* lines

Embryo injections were carried out by Rainbow Transgenic Flies, Inc. (Camarillo, CA, USA). The *vas*Cas9 and gRNA^sxl, βtub^ constructs were independently co-injected with Hsp70b-piggyBac transposase helper into freshly collected embryos of wildtype *D. suzukii*. G0 injected adults were outcrossed to *D. suzukii* wildtype flies and their G1 progeny were screened for the transformation event using the presence of the fluorescent marker with a Leica M165FC fluorescent stereomicroscope. Successfully transformed strains harboring the construct were introgressed over multiple generations to develop a stable mostly homozygous stock. The brightest line for the *vas*Cas9 and gRNA^sxl, βtub^, microinjections were identified and used for subsequent analysis.

### Insect rearing

Fly media used in this study consisted of a cornmeal/dry yeast/agar/molasses media with Tegosept. *D. suzukii* stock lines such as wildtype, *vas*Cas9, and gRNA^sxl, βtub^, were housed in small, modified cages (plant-based 32-oz soup bowl with 600 μm mesh on plastic lid) with a water source. These small cages were held in a Caron Insect Rearing Chamber at 20–21 °C with 65–70% relative humidity with a 12-hr light/dark cycle. To collect virgin females for experiments, wildtype females were sexed at the late pupae stage by looking for the absence of the male sex combs on the fore tarsus. Groups of 10–15 sexed female pupae were placed in individual *Drosophila* media vials for emergence. Sexed pupae were allowed to emerge and a second quality check for male flies were performed to ensure virginity of females. If a single male was detected in a sexed female vial, that vial was discarded. All mating experiments were performed in wide-vials filled with *D. suzukii* media.

### Fertility assessment

To confirm the fertility status of *vas*Cas9, gRNA^sxl, βtub^, pgSIT, and wildtype males, a series of outcrosses were performed with virgin wildtype females. All flies in this experiment were aged 2–3 days prior to setting up genetic crosses (Fig. 1C). The following crosses were set up: (1) five wildtype males with 15 wildtype virgin females; (2) five *vas*Cas9 males with 15 wildtype virgin females; (3) five gRNA^sxl, βtub^ males with 15 wild-type virgin females; (4) five sterile pgSIT males with 15 wildtype virgin females. Mating crosses were performed in triplicate. Duration of the experiment was three weeks and vials were flipped every three days or when bacterial film was present. The offspring from the genetic crosses were allowed to emerge then counted and compared.

### Female remating and offspring production

In order to test if sterile pgSIT males can induce a refractory period in wildtype females, the experiment was designed with the following negative control, positive control, and experimental treatments: The negative control consisted of 15 virgin wildtype females; The positive control consisted of 15 virgin wildtype females paired with 5 wildtype males; Treatment #1 consisted of an initial pair between 5 wildtype males and 15 virgin wildtype females. Wildtype males were then replaced with 5 pgSIT sterile males after the initial 7-day mating period; Treatment #2 consisted of an initial pair between 5 pgSIT males and 15 virgin wildtype females. pgSIT sterile males were then replaced with 5 wildtype after the initial 7-day mating period. The male switch described in treatments #1 and #2 was done to imitate a scenario where a wild female mates with one strain then encounters another male of a second strain. After the switch, male flies were not replaced throughout the duration of the experiment. The F1 offspring was screened under a Leica Stereoscope with fluorescence. An mcherry/GFP double pass filter was used to simultaneously see both GFP and RFP markers. The total number of offspring and average offspring counts were determined. All flies used were aged 2–3 days prior to preparing crosses.

### Testis dissection and imaging

To confirm the basis of sterility (by disruption of (βTub gene)), testis dissections were performed. Wildtype, gRNA^sxl, βtub^, *vas*Cas9, and pgSIT males were collected as pupae and allowed to emerge in vials. After emergence, the males were allowed to age for four days prior to testis dissections in 1x PBS buffer under a dissecting microscope. Testis from each strain were then visualized and imaged using the AMG EVOS FL Digital Inverted Microscope at 4X and 10X magnification. Fertile males were classified as having long spermatids spilling out of testis. Sterile were classified as lacking this mature sperm.

### Mated female reproductive tract dissection and imaging

To determine if male DNA could be detected in female reproductive tracts post mating, a series of matings and subsequent dissections were performed. Flies used in this experiment were collected and sexed as pupae to verify sex and virginity then aged for 2–3 days. Per treatment, 30 virgin wildtype females were paired with 15 males from gRNA^sxl, βtub^, *vas*Cas9, or pgSIT strains. As a negative control, virgin wildtype females were not paired with males. All flies, with the exception of the negative control, were allowed to mate with males for a total of five days to ensure all females had an opportunity to mate with a male. During this mating period, two females were randomly chosen on 1-, 3-, and 5- days post mating for ovary dissections and imaging. Ovaries were imaged using the View4K HD camera (Microscope Central) attached to the Leica M165FC stereomicroscope between 6.3X and 8.0X magnification. After the five-day mating period, 20 mated females were randomly chosen from each cross for dissections. Females were first immobilized on ice then transferred onto a microscope slide with one drop of 1x PBS. The female reproductive tract consisting of the ovaries, spermatheca, accessory glands, and the oviduct were dissected out and transferred to a tube containing 100 µL of QuickExtract™ DNA Extraction Solution (Lucigen, Cat.# QE0905T). For each treatment, 20 reproductive tracts were pooled for subsequent DNA extraction and molecular analysis.

### DNA extractions and PCR

DNA from reproductive tracts dissected from wildtype females that had mated with gRNA^sxl, βtub^, *vas*Cas9, or pgSIT males and virgin females was extracted following the manufactures’ protocol. Briefly, the samples underwent a heat treatment at 65 °C for 6 min followed by a heat kill step at 98 °C for 2 min. No purification step was performed. For PCR, 2 µL of 1:50 diluted DNA extract was used as template for PCR. The Kapa HiFi HotStart ReadyMix PCR kit (Roche, Cat. # KK2601) was used to amplify regions of the gRNA^sxl, βtub^ or *vas*Cas9 transgene (Supplemental Table 3). If mature sperm was transferred by mating, it could be molecularly detected in the female reproductive tracts of mated females. PCR products were run on a 2% agarose gel along with a 1 kb Plus DNA Ladder (NEB, Cat. N3200S). Gels were imaged using the Azure c400 (Azure Biosystems).

### Statistical analysis

In all experiments, at least three replicates were used to make comparisons between means. To determine significance, unpaired *t*-tests were used to compare experimental treatments to the control group. Comparisons were considered statistically significant with *p* < 0.05. The GraphPad Prism version 10.3.1 for macOS (GraphPad Software, San Diego) was used for these analyses.

## Electronic supplementary material

Below is the link to the electronic supplementary material.

Supplementary Material 1

Supplementary Material 2

## Data Availability

Data is provided within the manuscript or supplementary information files.
