# Supplementary material for: Dissecting the sterility phenotype in gene edited Drosophila suzukii pgSIT males
Source: Sci Rep. 2025 Jan 31;15:3903. doi: 10.1038/s41598-025-88598-w (PMC11785727; doi:10.1038/s41598-025-88598-w)
Supplement: Supplementary file 1 — Supplementary Material 1 [file 41598_2025_88598_MOESM1_ESM.docx]

**Supplementary Table**

Dissecting the sterility phenotype in gene edited *Drosophila suzukii* pgSIT males

**Authors**

Avery D. Witherbee^1^ and Stephanie Gamez^1^*

**Affiliations**

^1^Agragene Inc., St. Louis, MO, USA

*Correspondence to: Stephanie.gamez@agragene.com

**Supplemental Table 3. Primers used to amplify *vas*Cas9 and gRNA^sxl,βtub^ for detection.** Primers are used to detect a portion of the target gene for confirmation.

| **Target** | **Amplicon size** | **Orientation** | **Sequence 5’ to 3’** |
| --- | --- | --- | --- |
| *vas*Cas9 | 731 bp | Forward | GCCCCAAAGAAGAAGCGGAAGG |
|  |  | Reverse | AGCTGGGCGATCAGATTTTCCA |
| gRNA^sxl,βtub^ | 1.4 kb | Forward | CACTGCATTCTAGTTGTGGTTTGTCCA |
|  |  | Reverse | ATTGTCAGATCCGAGATCGG |
